# Effect of Myopic Femtosecond Laser-Assisted LASIK on Anterior Chamber Inflammation (Flare Values) and Corneal Endothelium: A Prospective before and after Study

**DOI:** 10.1155/2021/2395028

**Published:** 2021-11-26

**Authors:** Kemal Turgay Özbilen, Emre Altinkurt, Nihan Aksu Ceylan, Gizem Sayar Bilgin, Nilüfer Gözüm

**Affiliations:** Istanbul University, Istanbul Faculty of Medicine, Department of Ophthalmology, Istanbul, Turkey

## Abstract

**Purpose:**

This study aimed to investigate whether femtosecond laser-assisted LASIK (FS-LASIK) surgery causes inflammation in the anterior chamber and to analyze its effect on endothelial cells.

**Methods:**

This prospective, longitudinal study included left eyes of 30 patients (19 females) who had undergone FS-LASIK surgery due to myopia and myopic astigmatism. Endothelial cell density (ECD) and morphological measurements were performed using a specular microscopy, and laser flare photometry was used to measure the anterior chamber flare values on the day of surgery. iFS™ Advanced FS and VISX STAR S4-IR Wavescan Excimer Laser platforms were used. Flare measurements were repeated on the postoperative 1^st^ day and 7^th^ day and the 1^st^ and 3^rd^ months. The endothelial measurements were repeated in the 3^rd^ month.

**Results:**

Preoperatively, the mean flare was 5.59 ± 1.24 photons/ms; it was 6.49 ± 2.42 on the postoperative 1^st^ day, 5.87 ± 2.27 on the 7^th^ day, 5.68 ± 1.66 on the 1^st^ month, and 5.35 ± 1.24 on the 3^rd^ month. A significant difference was observed only between the preoperative and postoperative 1^st^ day flare values (*p*=0.047). The decrease in the ECD was clinically insignificant but statistically significant, with an average of 97.0 ± 209.9 cell count/mm^2^ (3.3%, *p*=0.017). However, there was no significant change in the coefficient of variation (*p*=0.448) and hexagonality (*p*=0.096). No significant correlation was found between the increase in the flare value on the postoperative 1^st^ day and variables. A significant correlation was found between the decrease in ECD and the preoperative ECD (*r* = 0.356, *p*=0.027).

**Conclusion:**

FS-LASIK caused minimal inflammation in the anterior chamber on only the 1^st^ postoperative day; additionally, a minimal decrease of cell count with no morphological changes were noted in the endothelial cells on postoperative 3^rd^ month. This trial is registered with NCT04899258.

## 1. Introduction

The femtosecond (FS) laser is a type of neodymium laser with a near-infrared ray wavelength (1053 nm). It can focus with very short pulses of 1/10^15^ of a second and make incisions by creating cavitation bubbles and a split interface during photodestruction of the corneal stroma [1, 2].

The use of FS in ophthalmology began in the early part of the 21^st^ century and marked a new era in corneal refractive surgery. It has been shown to create laser in situ keratomileusis (LASIK) flaps as well as or even better than mechanical microkeratomes (MMKs) [2–4]. FS-assisted LASIK (FS-LASIK) promises more predictable flaps and fewer flap complications, less ocular aberrations, better uncorrected visual acuity (UCVA), less variation in intraocular pressure, and less dry eye. However, complications, such as rainbow-like glare, haze, diffuse lamellar keratitis, and opaque bubble, cannot be ignored [2, 5–8].

The inflammation expected in corneal refractive surgery occurs in the corneal stroma; moreover, the inflammation that causes complications is often at the stromal interface [9–11]. However, it has been previously shown that refractive surgical procedures can have mechanical, photodestructive, and photochemical effects on the cornea and may stimulate apoptosis; inflammatory pathways and arachidonic acid metabolites can also cause inflammation in the anterior chamber [11–15].

Several studies have investigated the effect of FS laser-assisted cataract surgery on anterior chamber inflammation and corneal endothelium [16–18]. Furthermore, the effects of LASIK performed with MMKs, excimer laser with superficial ablation as photorefractive keratectomy (PRK), and even radial keratotomy and intrastromal rings on inflammation in the anterior chamber have been investigated [12, 19, 20]. Since FS-LASIK surgery is applied using both an FS laser and an excimer laser, we aimed to determine the direct and indirect effects of these applications on the corneal endothelium, the intensity of the inflammation, and the blood-aqueous barrier in the anterior chamber. To the best of our knowledge, the effects of FS-LASIK on anterior chamber inflammation have not been previously investigated. Therefore, we evaluated these effects using objective measurement methods, such as specular microscopy and laser flare photometry.

## 2. Materials and Methods

The left eyes of 30 patients who underwent FS-LASIK surgery at the Department of Ophthalmology, Istanbul University Istanbul Faculty of Medicine, between December 2019 and March 2020, were included in this prospective clinical study. Approval for the study was obtained from the institute's ethics committee (06.12.2019/20/1495), and signed informed consent was obtained from all the patients. The study was conducted in accordance with the ethical values stipulated in the Helsinki Declaration.

### 2.1. Inclusion Criteria

Patients older than 18 years of age, with a stable refraction in the previous year, eligible for LASIK surgery based on ophthalmological and topographical tests, and undergoing uncomplicated FS-LASIK surgery only because of myopia or myopic astigmatism were included.

### 2.2. Exclusion Criteria

Patients who did not show up for the postoperative follow-up were excluded. Patients with hyperopia, hyperopic, or mixed astigmatism, eyes found to be unsuitable for LASIK surgery, such as those with corneal ectasia, fruste keratoconus, and significant anterior or posterior elevation based on ophthalmological and topographic tests, and patients who underwent superficial ablation or other refractive surgery modalities or complicated LASIK were also excluded.

A complete ophthalmological examination was performed on the patients at each visit. Uncorrected distal visual acuity (UDVA) and corrected distal visual acuity (CDVA) were measured using decimal charts and were converted into logarithm of minimum angle of resolution (logMAR). Manifest and cycloplegic refractions were determined, and intraocular pressures were measured. Detailed anterior segment and fundus examinations were performed. Preoperatively, corneal topographies were obtained using Pentacam® HR (Oculus Optikgeräte GmbH, Wetzlar, Germany) and evaluated carefully on a quadruple refractive map to determine suitability for refractive surgery.

On the day of surgery, endothelial cell density (ECD), coefficient of variation (CV), and hexagonality measurements were performed by specular microscopy (Cellcheck SL, Konan Medical CA, USA). The anterior chamber flare values were measured by laser flare photometry (Kowa FM-700, Kowa company, Aichi, Japan); the average of three consecutive measurements was accepted as the flare value.

### 2.3. Surgical Procedure

Refractive surgeries were performed with an iFS™ Advanced Femtosecond Laser System (150 Hz, AMO GmbH, Ettlingen, Germany) and the VISX STAR S4-IR Wavescan Excimer Laser (AMO GmbH, Ettlingen, Germany). In all the patients, the flap diameter was set as 8.8–9.0 mm, the flap thickness was set at 100–110 microns, and the ablation amounts were recorded. Postoperatively, prednisolone sodium phosphate 1%, moxifloxacin 0.5%, and artificial tear drops (trehalose + Na hyaluronate + carbomer combination) were prescribed four times daily. Instillation was started immediately after the procedure. The antibiotic drop was discontinued at the end of the first week, and the steroid drop was tapered after the first week and used for three more weeks. The artificial tear drops were used for three months.

Flare measurements were repeated postoperative on the 1^st^ day, 7^th^ day, 1^st^ month, and 3^rd^ month. The endothelium measurements were repeated in the 3^rd^ month. The preoperative flare values and the flare values for postoperative 1^st^ day, 7^th^ day, 1^st^ month, and 3^rd^ month were compared. The preoperative and 3^rd^ month values of the endothelium measurements were compared.

### 2.4. Statistical Analysis

G^*∗*^ Power 3.1.9.6 software (Heinrich Heine University, Düsseldorf, Germany) was used to calculate the sample size. The primary and unique data investigated in the present study are the changes in anterior chamber flare value in the FS-LASIK applied eyes. However, since there is no study in the literature examining the flare change with FS-LASIK, we accepted as reference the study of Vita et al. [12], which reported the flare values after applying LASIK with MMK. When accepted the correlation between dependent groups (before vs. after) is 0.5, the *α* error is 0.05 and the power (1-*β*) is 0.95, the result; required total sample size was found to be 28 eyes. So, the study was planned at least with 30 subjects.

SPSS version 22 software program was used for statistics. A paired *t*-test was used in the first test-last test situations and the cross-comparisons. Pearson correlation analysis and linear regression analysis were performed to examine the relationship between the parameters and these changes. A *p* value less than or equal to 0.05 was considered statistically significant.

## 3. Results

A total 30 patients' left eyes (19 female and 11 male) were evaluated. The mean age of the patients was 24.97 ± 3.6 years (range: 21–37 years). The mean central corneal thickness was 564.7 ± 33.3 microns (range: 506–649 microns), the mean ablation amount was 65.93 ± 28.61 microns (range: 30–133 microns), and the mean flap diameter was 8.90 ± 0.10 millimeters (range: 8.8–9.0 millimeters).

As expected, UDVA increased significantly, and the mean spherical, cylindrical, and spherical equivalent refractive errors decreased significantly in all the eyes, postoperative. The mean CDVA did not change. The patient characteristics are given in Table 1.

**Figure 1:**
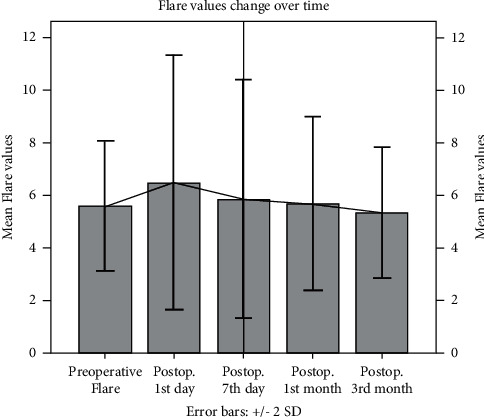
Change in flare values (photons/ms) over time.

The average flare value was 5.59 ± 1.24 photons/ms, preoperatively; it was 6.49 ± 2.42 photons/ms on the postoperative 1st day, 5.87 ± 2.27 photons/ms on the 7^th^ day, 5.68 ± 1.66 photons/ms in the 1^st^ month, and 5.35 ± 1.24 photons/ms at the 3^rd^ month. A statistically significant difference in the mean flare values was observed only between the preoperative and postoperative 1^st^ day values (*p*=0.047). However, this difference was minimal (mean dif = 0.90 ± 2.38 photons/ms). There was no significant difference in the cross-comparison of flare values of the other postoperative visits. The changes in average flare values are shown in Figure 1, and the cross-comparisons are given in Table 2.

Although no significant abnormality in endothelium functions was observed clinically, some statistically significant changes were observed in specular microscopy measurements. The mean ECD was decreased with an average of 97.0 ± 209.9 (3.3%) cell count/mm^2^ (*p*=0.017). While, evaluating endothelial cell morphology showed that both the mean CV and hexagonality did not change significantly (*p*=0.448 and *p*=0.058, respectively). The changes in corneal endothelial cells are given in Table 3.

The relationships between the parameters and the increase in the flare value on postoperative 1^st^ day and the decrease in ECD were investigated using linear regression analysis. A significant correlation was found only between the decrease in ECD and the preoperative ECD (*r* = 0.356, *p* < 0.027). No significant correlation was found between the increase in the flare value on postoperative 1^st^ day and parameters. The correlations are given in Table 4.

## 4. Discussion

The present study found a statistically significant but minimal increase in flare value on the postoperative 1^st^ day after FS-LASIK; however, no significant difference was found between the preoperative values and the values for the other postoperative visits. We found no correlation between the flare elevation on the postoperative 1^st^ day and any parameters.

However, in other studies, on anterior chamber inflammation related to other corneal refractive surgeries, such as LASIK performed with MMKs, El Harazi et al. [19] investigated anterior chamber inflammation after LASIK was performed with MMKs and reported high flare values only on the 1^st^ postoperative day; they did not find a correlation for this elevation. The values returned to normal on the 7^th^ day postoperative. Sen et al. [21] reported that the flare values increased in the first hours after performing the LASIK procedure to correct high astigmatism in patients treated with penetrating keratoplasty before surgery, but decreased to normal levels even on the 1^st^ day. Pisella et al. [20] reported high and prolonged inflammation after LASIK (performed with MMKs); they reported that the increase in the amount of flare was greater on the 1^st^ day postoperative, especially in the eyes undergoing LASIK; however, they also reported that the flare values returned to normal levels on the 7^th^ day postoperative in PRK, but this period was prolonged in LASIK and no significant increase in a flare was observed at any time in patients who underwent intrastromal ring implantations. Pérez Santonja et al. [22] reported that, after LASIK with MMKs, there was no increase in the flare values on the 1^st^, 3^rd^, and 7^th^ days, postoperative. Interestingly, they also reported that the flare values decreased after the 2^nd^ week in comparison to the preoperative value, and they only reached typical values in the 3^rd^ month, postoperative.

Our findings suggest that FS-LASIK does not cause a significant increase in inflammation in the anterior chamber, and it does not cause severe damage to the blood-aqueous barrier. The minimally elevated flare values on the 1^st^ day and the near-normal values of consecutive postoperative visits may be related to the use of topical steroid drops instilled immediately after surgery and easily controllable mild inflammation. As mentioned above, in the literature, the presence of more pronounced inflammation findings after LASIK performed with MMK in comparison to the values we found with FS-LASIK suggests that MMK is more associated with inflammation than FS-LASIK. To the best of our knowledge, no previous study has investigated anterior chamber inflammation related to FS-LASIK. Our study is the first to report on anterior chamber flare values after FS-LASIK.

We found clinically insignificant but statistically significant mild changes in corneal endothelium. Such as a decrease of 3.3% in ECD occurs without a significant change in cell morphology at the end of the 3^rd^ postoperative month for patients undergoing FS-LASIK. However, a significant correlation was found between the endothelial loss and only the preoperative ECD. In contrast, Tomita et al. [23] reported no significant changes in ECD and its morphologies three months after FS-LASIK surgery was performed with two different FS platforms, and no difference was found between the two platforms. In their series of 21 patients comparing FS-assisted LASIK vs. MMKs, Klingler et al. [24] also reported no significant change in ECD in both techniques at the end of a 5-year follow-up. In the literature, it has been reported that there is no significant decrease in ECD after PRK [25, 26]. Collins et al. [27] reported that they did not detect any change in the endothelium in the 3-year follow-up after LASIK was performed with MMKs. Durrie et al. [28] compared PRK with thin-flap LASIK and reported no significant changes in the ECD at the 3^rd^ postoperative month with both techniques and no difference between the two techniques. However, similar to us, a recently published study by Shaaban and Badran [29] reported a statistically significant decrease in ECD and a mild change in endothelial morphology, although clinically insignificant in both FS-LASIK and small incision lenticule extraction (SMILE).

The effect on the endothelial cells may be related to the heat energy generated by the shock waves that occur when the flap is created with the femtosecond laser rather than the excimer laser ablation. Because our study consists of a cohort with a performed low level of myopic ablation, no correlation was found between ablation amount and endothelial loss. Although FS-LASIK may not affect endothelial cells in all patients, it may affect some susceptible patients. This respects that it is essential to be careful in cases with endothelial abnormalities, such as endothelial dystrophy because, in the literature, there are case reports of cornea guttata or Fuchs endothelial dystrophy being decompensated after LASIK [30, 31].

This study has some limitations. It only included a small number of cases, it did not include a control or comparison group, and due to the risk of diffuse lamellar keratitis and regression, steroid drops were used in all patients in postoperative treatment. Moreover, the follow-up time was insufficient to enable a more realistic observation of the change in corneal endothelium. We are also aware that there may be contradictory results technically between the consecutive measurements in specular microscopy measurements, which may affect our findings.

Furthermore, FS-LASIK is a two-step procedure, and it is difficult to predict from which step the effect originates. A comparison of FS-LASIK with LASIK performed with MMK, or with a procedure performed with only FS, such as SMILE, or a method with no flap, such as PRK, may provide some more helpful information. Nevertheless, in PRK, LASIK, and SMILE, the stromal depth at which the incision and ablation are made is different, which can change their effect. However, the lack of a significant change in the amount of flare reduces the need for a comparison group in order to detect which step is associated with flare change. In addition, since it is well-defined in the literature that LASIK performed with MMK does not significantly reduce the mean ECD, it may be considered that this decrease may be related to the FS laser rather than the excimer laser. The advantages of the present study are that it is a prospective, longitudinal study, and objective measurement methods were used.

## 5. Conclusions

According to the present study's findings, FS-LASIK does not cause severe inflammation in the anterior chamber, and it does not significantly increase the amount of flare except for minimal elevation on the 1^st^ postoperative day. In this respect, it offers a safe profile in terms of postoperative intraocular inflammation. To the best of our knowledge, this study is the first to evaluate anterior chamber inflammation and report flare values in FS-assisted LASIK refractive surgery. Additionally, FS-LASIK may affect the corneal endothelium. Therefore, if performing FS-LASIK on a susceptible patient group at risk for endothelial cells damage (e.g., family history or signs of corneal guttata or history of previous intraocular surgery or ocular trauma), it may be beneficial to keep this finding in mind.

## Figures and Tables

**Table 1:** Patient characteristics.

Variables/means	Preoperative	Postoperative 3^rd^ month	*P* value (*t*-test)
UDVA (logMAR)	1.13 ± 0.52	−0.018 ± 0.08	<0.0001
CDVA (logMAR)	−0.020 ± 0.041	−0.040 ± 0.072	0.206
Spherical R. Err (D)	−3.30 ± 1.84	−0.050 ± 0.31	<0.0001
Cylindrical R. Err (D)	−0.98 ± 0.91	−0.34 ± 0.28	0.001
Spherical equivalent R. Err (D)	−3.79 ± 1.86	−0.233 ± 0.28	<0.0001

UDVA, uncorrected distance visual acuity; CDVA, corrected distance visual acuity; R. Err, refractive error; D, diopters.

**Table 2:** Cross-comparison of the flare values, paired *t*-test (*p* values).

Flare value	Preoperative	1^st^ day	7^th^ day	1^st^ month	3^rd^ month
Preoperative		**0.047**	0.496	0.766	0.276
1^st^ day	**0.047**		0.301	0.099	0.052
7^th^ day	0.496	0.301		0.699	0.242
1^st^ month	0.766	0.099	0,699		0.202
3^rd^ month	0.276	0.052	0.242	0.202

The bold values represent *p* < 0.05.

**Table 3:** Endothelial cell assessment: ECD and morphological features.

Variables	Preoperative	Postoperative 3^rd^ months	*P* value (paired *t*-test)
Endothelial cell density (ECD) (cell/mm^2^)	2915.1 ± 199.4 (2500–3311)	2818.6 ± 232.3 (2463–3326)	**0.017**
Coefficient of variation (CV) in cell size	27.4 ± 2.5 (24–33)	27.6 ± 2.5 (23–34)	0.448
Hexagonality (%)	59.0 ± 3.6 (52–66)	58.5 ± 3.5 (52–65)	0.096

The bold value represents *p* < 0.05.

**Table 4:** Correlations for flare elevation on the postoperative 1^st^ day and endothelial cell loss (linear regression analysis).

Parameters	Correlations between flare elevation and variables		Correlations between endothelial loss and variables	
	Pearson' *r*	*P*	Pearson' *r*	*P*
Preoperative UCVA	0.216	0.126	0.010	0.479
Preoperative CDVA	−0.018	0.463	0.158	0.203
Preoperative spherical R. Err	−0.139	0.145	0.128	0.249
Preoperative cylindrical R. Err	0.114	0.275	0.209	0.134
Preoperative spherical. equivalent	−0.221	0.120	0.178	0.173
Preoperative CCT	0.050	0.396	0.140	0.231
Ablation dept	0.227	0.114	0.133	0.242
Age	0.083	0.331	−0.127	0.252
Preoperative hexagonality	−0.181	0.169	0.151	0.212
Preoperative coefficient of variation	0.120	0.264	−0.037	0.422
Preoperative flare value	−0.226	0.116	0.239	0.101
Preoperative endothelial count	0.044	0.409	0.356	**0.027**

CCT, central corneal thickness; UDVA, uncorrected distance visual acuity; CDVA, corrected distance visual acuity; R. Err, refractive error. The bold represents *p* <0.05.

## Data Availability

The datasets generated during and/or analyzed during the current study are available from the corresponding author upon request.
